# Healthcare practitioners’ views of comprehensive care to mental healthcare users in a community setting

**DOI:** 10.4102/curationis.v45i1.2349

**Published:** 2022-11-23

**Authors:** Ontlotlile I. Mpheng, Belinda Scrooby, Emmerentia du Plessis

**Affiliations:** 1 School of Nursing Science, Faculty of Health Sciences, North-West University, Mahikeng, South Africa; 2 School of Nursing Science, Faculty of Health Sciences, North-West University, Potchefstroom, South Africa

**Keywords:** community, healthcare practitioners, comprehensive care, mental healthcare, mental healthcare users, rural, stakeholders

## Abstract

**Background:**

Comprehensive care means ensuring quality services, protecting rights, promoting available social services and using protocols and standards that emphasise quality assurance for all mental healthcare users (MHCUs). It also involves advocacy, early detection and rehabilitation, as well as encouraging appropriate patient-centred care to ensure adequate psychiatric care. However, according to research, there is a vacuum in the provision of comprehensive mental healthcare to MHCUs. As a result, there is an immediate need to consult healthcare providers on providing comprehensive community-based care to MHCUs.

**Objectives:**

The purpose of this study was to explore and describe the views of healthcare practitioners on the aspects that hinder providing comprehensive care for MHCUs, the role players needed to execute comprehensive care and what can be done to improve comprehensive care for MHCUs in the community setting in one of the subdistricts of the North West province (NWP), South Africa (SA).

**Method:**

A qualitative research design that was exploratory, descriptive and contextual was adopted. The healthcare practitioners that took part in the study were chosen through purposive sampling. The sample size was established through data saturation, and 19 telephonic semistructured individual interviews were held with registered nurses and one medical doctor. Tesch’s eight steps were used to analyse the data.

**Results:**

The four main themes identified were: (1) healthcare practitioners’ understanding of comprehensive care to MHCUs, (2) factors hindering comprehensive care to MHCUs, (3) stakeholders needed for providing comprehensive care to MHCUs and (4) suggestions for improving comprehensive care to MHCUs.

**Conclusion:**

Healthcare practitioners in the community advocate for the need for comprehensive psychiatric treatment. They are of the view that greater coordination of psychiatric services will improve mental treatment and minimise relapse in MHCUs. To sustain integrated psychiatry, stakeholders and other psychiatric programmes must be included.

**Contribution:**

The findings and conclusions of this study indicated that improvement is needed in mental healthcare in general, and all relevant aspects to improve comprehensive care among MHCUs in a community setting should be given full attention.

## Introduction

Although mental healthcare services are available in communities where mental healthcare users (MHCUs) reside and collect their mental health medication, research shows that there is still a lack of human resources, for example, psychologists and psychiatrists, and the necessary comprehensiveness in mental health services (Petersen et al. 2016). Comprehensive mental healthcare appears to be ideal, but in reality, there are frequently insufficient multidisciplinary team (MDT) members in the community with the requisite abilities to provide this care to MHCUs (Alburquerque-Sendin et al. 2018; Yesuku-Udechuku et al. 2015).

Comprehensive care should include both human resources and the necessary mental healthcare services for complete care. The Mental Health Atlas (MHA) provides a framework for assessing and improving mental healthcare services, including the various components of a comprehensive mental healthcare system (MHA 2014). Comprehensive mental healthcare services include the protection of quality services; promoting available social and psychological services; prevention methods; availability of treatment; and availability of healthcare practitioners (that is, registered nurses, as well as a medical doctor, psychiatrist, social worker and psychologist) (MHA 2014; Petersen et al. 2016). Comprehensive community care for MHCUs is critical because to be in a decent mental state, they require attention from MDTs.

Factors obstructing comprehensive care of MHCUs are outlined, such as the shortage of psychiatric services (including ensuring quality services) and the inadequate protection of rights. Barriers also include the need for better promotion of available social services, early detection, patient-centred care and building of rehabilitation services (Madikizela 2017; Petersen et al. 2016). Other obstacles to providing comprehensive mental healthcare include mental health stigma and the lack of time and necessary resources to support and treat patients (Loeb et al. 2016).

Owing to the unavailability of comprehensive care, MHCUs in the community relapse (Petersen et al. 2016). A lack of both dedicated mental health practitioners and allotted funds, and insufficient accountability for quality service delivery, are all signs that mental health treatment in community settings is still a matter of concern (Ellie 2020). Regardless of the implementation of the Programme for Improving Mental Healthcare (PRIME) guideline for community mental healthcare, many challenges remain, such as the lack of dedicated mental healthcare practitioners and inadequate funds (Madikizela 2017).

There is a need for improvement in the provision of comprehensive mental healthcare for MHCUs (Acharya et al. 2017). This need is especially noted in rural communities in South Africa (SA) (Vergunst 2018), including in the Mahikeng subdistrict of the North West province (NWP). In addition, healthcare practitioners in community settings can play an important role in getting closer to the ideal form of comprehensive care (Schneider et al. 2016). As a result, it was identified that there was a need to explore and describe healthcare practitioners’ perspectives on providing comprehensive care to MHCUs in a community setting in one of SA’s rural provinces. Such research would raise knowledge and awareness of how mental healthcare services can be improved to help MHCUs have a better quality of life in the community.

### Research objective

The purpose of this study was to explore and describe the views of healthcare practitioners on the following:

- providing comprehensive care
- what hinders comprehensive care for MHCUs
- who is needed to execute comprehensive care
- what can be done to improve comprehensive care for MHCUs in the community setting in one of the subdistricts of the NWP, SA.

## Research methods and design

### Research design

In this study, a qualitative, exploratory, descriptive and contextual design was utilised (Polit & Beck 2017). This research design allowed the researcher to explore and describe the views of healthcare practitioners on providing comprehensive care regarding aspects that hinder comprehensive care for MHCUs, role players needed to execute comprehensive care and what can be done to improve comprehensive care for MHCUs in the community setting in one of the subdistricts of the NWP, SA. This research design was deemed appropriate because it allowed for in-depth perceptions of healthcare practitioners on providing comprehensive care to MHCUs.

### Setting description

Data were collected from five community health centres (CHCs) and four clinics in a Mahikeng rural community (Mahikeng subdistrict) in one of SA’s provinces, known as NWP. The MHCUs go to these facilities for mental health treatment and after being discharged from the mental health hospital for continuity of care.

### Population and sampling

The population consisted of MDT members such as professional nurses, medical practitioners, psychologists, social workers and other therapists. Participants were recruited to the study based on the inclusion criteria of having three or more years of experience working with MHCUs and being members of the MDT. Purposive sampling was used to select participants who were the best candidates for understanding the research phenomenon. Nineteen professional nurses and one medical practitioner agreed to participate and comprised the research sample (Polit & Beck 2017). Additional members of the MDT were not available in the communities owing to coronavirus disease 2019 (COVID-19) restrictions.

The recruitment process was aided by a mediator (district manager) and facility managers. Following the facility managers’ identification of potential participants, the first author gave them a brief overview of the study; each participant then gave their initial verbal informed consent to participate. The first author then offered definitions of terms, defined the purpose of the research and after 3–5 days, the first author again explained informed consent to the participants via WhatsApp. Participants who agreed to participate signed informed permission forms in front of the facility manager; the forms were then scanned and returned to the first author.

### Data collection

Individual telephonic semistructured interviews were used. This allowed flexible communication between the researcher and participants. Such interviews were chosen because they allowed the first author to use open-ended questions, allowed the participant to supply the most relevant replies and enabled both the researcher and the participants to adhere to the COVID-19 regulations in place at the time of the study. The interview questions included:

- In your view, what does comprehensive mental healthcare entail?
- In your opinion, what factors hinder comprehensive care for MHCUs in the community setting?
- Who do you think is needed to provide comprehensive care for MHCUs in the community setting?
- What do you think can be done to improve comprehensive care for MHCUs in the community setting?
- Any other comments on comprehensive care for MHCUs?

Emans’s communication skills (Emans 2019) were applied, such as maintaining a neutral attitude and the probing approach. The researcher asked probing questions so the participants could answer in more detail. The interviews lasted between 30 min to 1 h, and the researcher used audio recorders to record all the interviews.

### Data analysis

To detect patterns and relations based on the data, the data were organised in an orderly, coherent manner. The qualitative data were analysed using the eight steps of Tesch’s coding process, comprising assimilation and synthesis of the data, emerging into condensed themes and subthemes (Mphasha, Mothiba & Skaal 2022). The data were shared with an independent co-coder for independent data analysis, and a consensus-seeking discussion of the emerging themes and categories was held. The steps for data analysis include: verbatim transcription of the raw data; cursory scanning of the raw data; attentive reading of the transcribed data to get a general understanding of the data and be able to reflect on their meanings; omitting irrelevant details and getting the data ready for coding; and properly generating and organising the concepts, categories and themes for clarity and validation (Mphasha et al. 2022). This was carried out to make sure that the data collected reflected the participants’ real thoughts.

### Rigour

Trustworthiness was ensured following the criteria outlined by Guba and Lincoln (1985), namely: credibility, dependability, transferability and confirmability. The researcher maintained prolonged engagement with participants for at least 2 months in the study until data collection was achieved. During data collection, participants were given ample time to share their perceptions regarding the provision of comprehensive care to MHCUs in a community setting. To ensure credibility, the study was examined by internal and external examiners. To ensure dependability, a thick description of the research study was maintained to ensure the possibility of the study being repeated in a different context. The results were confirmed by nine individuals who carried out member-checking; this ensured the confirmability of the study. Raw data were used to guarantee confirmability, and notes were taken and retained at a secure location for auditing purposes.

### Ethical considerations

Written legal authorisation was obtained from both the North-West Provincial Department of Health and the gatekeeper (primary healthcare manager) of the health facilities where the researcher collected the data. Ethical approval was obtained from the Research Ethics Committee of the relevant Faculty of Health Sciences (ref. no. NWU-00323-20-A1). Participants were given an in-depth description of the research. Informed signed consent forms were completed by the participants as proof of voluntary and informed participation in this study. Privacy and confidentiality were maintained throughout the research study.

## Results and discussion

The healthcare practitioners shared their perceptions regarding the provision of comprehensive care to MHCUs in a community setting. As summarised in Table 1, the themes emerging from data analysis included: (1) healthcare practitioners’ views of comprehensive care for MHCUs, (2) factors hindering comprehensive care for MHCUs, (3) the need for stakeholders to provide comprehensive care to MHCUs and (4) suggestions for improving comprehensive care for MHCUs. Subthemes could be identified for each of these themes.

**TABLE 1:** Healthcare practitioners’ views regarding providing comprehensive care to mental healthcare users in a community setting.

Theme 1	Theme 2	Theme 3	Theme 4
Healthcare practitioners’ understanding of comprehensive care to MHCUs: Comprehensive approach to mental healthcare, treatment and rehabilitation services Provision of psychiatric treatment	Factors hindering comprehensive care to MHCUs: Lack of resources: ■ Shortage of nursing staff ■ Financial constraints ■ Lack of psychiatric medication Lack of support: ■ Lack of family support ■ Lack of support from non-nursing staff Poor counselling skills Stigma attached to mental illness	Stakeholders needed for providing comprehensive care to MHCUs: Involvement of family members Involvement of community members Involvement of multidisciplinary team (MDT) members	Suggestions for improving comprehensive care to MHCUs: Conducting mental health education Conducting awareness campaigns Establishment of support groups Participating in community enrichment projects Availability of psychiatric medication Employment of more healthcare professionals More in-service training for healthcare practitioners

*Source*: Sibalala, O.I., 2021, ‘Healthcare providers’ perceptions of providing comprehensive care to mental health care users in a community setting’, Master dissertation, North West University, Potchefstroom, viewed n.d., from http://hdl.handle.net/10394/37738

MHCU, mental healthcare user.

The next paragraphs provide an overview of the themes and their subthemes. After each theme is examined, excerpts from the participant’s quotations and a discussion of existing literature are provided.

### Theme 1: Healthcare practitioners’ understanding of comprehensive care to mental healthcare users

The following subthemes emerged from the theme ‘Healthcare practitioners’ understanding of comprehensive care to mental healthcare users’: comprehensive approach to mental healthcare, treatment and rehabilitation services; and provision of psychiatric treatment.

#### Subtheme 1.1: A comprehensive approach to mental healthcare, treatment and rehabilitation services

Participants described comprehensive care as nonmedical treatment, such as psychological, clinical, mental, social and rehabilitative services that are provided by healthcare practitioners. They further stated that comprehensive care includes medical treatment with psychiatric medication that is always available to MHCUs in community settings. Participants reiterated that MDT members, the community and the family are all involved in comprehensive care:

‘In my understanding I think it should be taking care of psychiatry patients in community and clinical settings, as well in all spheres of life, that’s how I understand it … In all spheres of life meaning there are levels of understanding as well psychologically, clinically, mentally, and socially.’ (Participant D, female, registered nurse [RN])

The literature supports comprehensive care as meeting the following goals: developing community and home-based rehabilitation care; providing early detection and follow-up care; promoting available social support services; and attending to mental healthcare users’ biological, psychological, social, environmental and economic needs (Acharya et al. 2017; Alburquerque-Sendin et al. 2018).

#### Subtheme 1.2: Provision of psychiatric treatment

Participants feel that psychiatric treatment should always be available as part of comprehensive psychiatric care because when MHCUs are stable, everyone is safe, including patients’ lives, since relapsing MHCUs do not generally engage in hazardous behaviours. According to the participants, psychiatric treatment for MHCUs should be available at all times, and assistance for compliance should be maintained since it prevents relapse in MHCUs. Participants went on to remark that the availability of this treatment is critical not just for the well-being of the MHCUs but also for others around them, since when they are stable, everyone else is safe:

‘In my opinion, it means giving all healthcare to psychiatry patients by giving psychiatry treatment to them.’ (Participant C, female, RN)

The literature also suggests that psychiatric treatment should meet minimum clinical practice standards and that effective psychiatric treatment should keep MHCUs stable and reduce community indices of relapse or psychological distress, as well as suicide (Edworthy, Sampson & Völlm 2016; Mulder, Rucklidge & Wilkinson 2017).

### Theme 2: Factors hindering comprehensive care to mental healthcare users

The following subthemes emerged from the theme ‘Factors hindering comprehensive care to MHCUs’: a lack of resources, a lack of support, poor counselling skills and stigma attached to mental illness.

#### Subtheme 2.1: Lack of resources: Shortage of nursing staff, financial constraints and lack of psychiatric medication

Most participants thought that the shortage of mental health medication is a serious problem, as the medication is regularly out of stock at the pharmacies of healthcare institutions. As a result, many MHCUs end up defaulting on their treatment and relapsing. According to the participants, nursing staff shortages, budgetary limits for mental healthcare and psychiatric pharmaceutical shortages are among the challenges that healthcare practitioners are concerned about when it comes to comprehensive care:

‘Because of the shortage itself, it sometimes becomes very difficult for us to maintain a list of people that we have in the community and actually make sure that they adhere to compliance half the time we don’t have the means we don’t have the numbers to go out in the community.’ (Participant A, female, RN)

The literature also suggests that a global shortage of nurses is a big problem (Shamsi & Peyravi 2020). Further obstacles include a shortage of healthcare personnel who are professionally trained in psychiatric or mental healthcare; this then leads to staff burnout (Kohrt et al. 2018; Roets, Poggenpoel & Myburgh 2018).

On other aspects, participants shared that MHCUs do not adhere to follow-up dates owing to financial constraints. A participant’s inputs states:

‘Some of the people default because they can’t afford to go to [*name of hospital withheld due to anonymity*] for argument’s sake to fetch medication on a regular monthly basis.’ (Participant A, female, RN)

Similar studies confirm that low-income MHCUs experience psychological stress and find it difficult to access healthcare (Mulder et al. 2017; Schneider et al. 2016).

The other challenge participants agreed upon is a lack of medication, since the pharmacy at the mental healthcare institution is always out of stock of psychiatric medication:

‘Shortage of medication because sometimes they come and there is no medication because mostly of their scheduled medication are not kept in the facility.’ (Participant E, female, RN)

The literature confirms that inadequate distribution of psychiatric medication results in a significant treatment gap (Kohrt et al. 2018; Sidana 2018).

#### Subtheme 2.2: Lack of support: Lack of support from family and non-nursing staff

It was the view of the participants that a factor that hinders comprehensive mental healthcare is the lack of family support. They believed that most MHCUs do not adhere to follow-up dates as they are sometimes not mentally healthy and tend to forget, resulting in relapse. Mental healthcare users need support from family members to remind them to attend follow-up appointments:

‘Lack of support by the family at home if the psychiatry patients does not have enough support from the family at home it will be difficult for them to adhere in their medication.’ (Participant B, female, RN)

Literature confirms that when there is a lack of family support, MHCUs tend to relapse, limiting their access to comprehensive mental healthcare (LeCloux et al. 2016).

Another factor that hinders comprehensive mental healthcare is a lack of support from non-nursing staff, namely administration staff and emergency medical personnel. Mental healthcare users require specialised mental healthcare attention, and the limited availability of such MDT members limits comprehensive care and causes weariness in the staff who are available:

‘They highlight the fact that they called the police they have called the Emergency Medical Response Service but none of them are willing to come and assist unless they bring the patient to the clinic and sometimes is actually a serious exercise to the family.’ (Participant A, female, RN)

Authors support the idea that comprehensive care is hindered as nurses experience stress as a result of overwork and having to juggle administrative responsibilities (Roets et al. 2018). Nurses expressed burnout and stress as a result of a lack of assistance, as they are required to perform domestic as well as clerical chores (Joubert & Bhagwan 2018).

#### Subtheme 2.3: Poor counselling skills

Participants stated that they do not have specialisation in psychiatric or mental healthcare, that their counselling abilities are insufficient and that they are unable to reach the depths of MHCUs in need of psychological support:

‘I think the other one is poor counselling skills when it comes to providing care to psychiatry patients most healthcare professionals have poor counselling skills.’ (Participant C, female, RN)

According to the literature, poor counselling skills are still a matter of concern in the healthcare setting. Because of a lack of training, capacity building and skills, healthcare practitioners (e.g. nurses) are not sufficiently trained to manage psychiatric problems (Koposov et al. 2017).

#### Subtheme 2.4: Stigma attached to mental illness

Participants agreed that MHCUs in community settings are treated unfairly simply because they have a psychiatric condition and that they do not have the freedom to live their lives. According to the participants, members of the community do not adequately comprehend mental health. Furthermore, the services where the MHCUs are treated are not therapeutic, and as a result, MHCUs are not fully accepted as they are treated differently:

‘With psychiatry care, there is sort of a stigma to the condition that the user can experience which is not necessary really.’ (Participant F, female, RN)

According to the research, stigma leads MHCUs to decline follow-up therapy for fear of being judged, and family members are scared to express their views about their loved ones’ mental health and other concerns because they are afraid of being judged (Chan, Wong & Chien 2018; Kohrt et al. 2018).

### Theme 3: Stakeholders needed for providing comprehensive care to mental healthcare users

The theme, which is divided into three subthemes, focuses on the stakeholders required to provide comprehensive treatment to MHCUs.

The following subthemes emerged from the theme ‘Stakeholders needed for providing comprehensive care to MHCUs’: involvement of family members, involvement of community members and involvement of MDT members.

#### Subtheme 3.1: Involvement of family, community members and multidisciplinary team members

When MHCUs come in for a review, they require not just medical treatment but also psychological, physical, social and financial assistance. The participants felt that giving care to MHCUs requires a diverse group of healthcare practitioners. The participants believed that involving community members, family members and MDT members in caring for MHCUs or promoting mental healthcare will help improve the image and standard of mental healthcare because healthcare practitioners and members of the community will be working together through community educational programmes. They will also be sharing knowledge of how community values and principles are governed, and the MHCUs will benefit from this:

‘Families must be involved, there is nothing that can be done without families of psychiatry patients.’ (Participant E, female, RN)

According to literature, the primary source of treatment for psychiatry patients is their family members (Kohrt et al. 2018):

‘It helps a lot with regard to the family supporting the user and the community in terms of neighbours in the streets.’ (Participant A, female, RN)

The reason for community involvement, according to literature, is that if primary care services are not available, MHCUs will at least know where to get aid or care in the community (Kohrt et al. 2018):

‘You know when it comes to a psychiatry patient, I will say MDT they are needed to provide the comprehensive … starting from the psychiatrist, the psychologist, the social workers, and even the professional nurse.’ (Participant B, female, RN)

Different authors advocate for the inclusion of MDT members. The MDT members’ involvement is critical in providing comprehensive care in a community context. The areas of practice, interests, commitment, confidence, skills and efficacy of healthcare practitioners in evaluating and treating psychiatry issues vary (Olfson 2016; Schneider et al. 2016).

### Theme 4: Suggestions for improving comprehensive care to mental healthcare users

The following subthemes emerged from the theme ‘Suggestions for improving comprehensive care for MHCUs’: conducting mental health education, conducting awareness campaigns, establishment of support groups, participating in community enrichment projects, availability of psychiatric medication, employment of more healthcare professionals and more in-service training for healthcare practitioners.

#### Subtheme 4.1: Conducting mental health education and awareness campaigns

According to the participants, mental health initiatives might provide awareness to members of the community and could help to eliminate stigma. A majority of the participants agreed that providing mental health education and community awareness campaigns may help people – particularly community members – have a better understanding of mental health concerns:

‘Community psychiatry education with regard to the recent mental illness that are there that arise and that concern us as a community.’ (Participant A, female, RN)

Mental health education is seen as a critical component of effective mental health treatment. Health technologies (including instructional procedures and information) are required to maintain the supply of comprehensive services (Campos, Bezerra & Jorge 2018; Kohrt et al. 2018):

‘I think awareness campaign for our community because when it comes to psychiatry there is a lot of stigma so if our community can be educated with regards to what is psychiatry.’ (Participant C, female, RN)

The literature supports the concept that creating mental health awareness will assist in spreading more details regarding mental health in communities; such support will also contribute to comprehensive care (Kohrt et al. 2018; Sidana 2018).

#### Subtheme 4.2: Establishment of support groups

The need for support groups for MHCUs in the community was expressed by the majority of the participants, as this will demonstrate to the MHCUs that they are not alone. Participants shared that support groups could assist MHCUs in sharing their problems and in giving one another emotional support and advice; in turn, it also contributes to improving their mental health:

‘I wish people can actually take it a fact that support groups are there to support them, to teach them, to help their knowledge.’ (Participant A, female, RN)

Support groups are encouraged in literature also. Participants in other studies have talked about how support groups helped them deal with stress and improve mental health, self-care and work performance (Kahn et al. 2016; Reiser, Murphy & McCarthy 2016).

#### Subtheme 4.3: Participating in community enrichment projects

According to the participants, extramural activities should be implemented in community settings, thus allowing MHCUs to refresh their minds and keep themselves busy. Participants proposed that rehabilitation programmes should use extramural activities because they believed that these would help to rejuvenate MHCUs’ minds.

One of the participant stated:

‘They must do something in the community like playing soccer or doing certain chores like removing the grass, gardening, and other stuff.’ (Participant B: female, RN)

Family activities, social and independent living, skills training, medication adherence support groups, dealing with stigma, employment opportunities and income generation (e.g. gardening projects, self-help groups and life skills programmes), which lead to improved social inclusion, are all necessary for ensuring community enrichment (Kohrt et al. 2018).

#### Subtheme 4.4: Availability of psychiatric medication

Participants felt that having psychiatric medication available at healthcare facilities will allow smoother care since MHCUs will not have to go to the hospital for treatment; further, the risk of relapse by an MHCU will thereby be reduced:

‘Government need to put measures into place to make sure that medication to psychiatry patients is always available.’ (Participant A, female, RN)

Literature supports the assertion that the availability of affordable and appropriate medication at community-based public mental healthcare facilities should be ensured (Kohrt et al. 2018; Sidana 2018).

#### Subtheme 4.5: Employment of more healthcare professionals

Comprehensive care can be maintained through having enough human resources. Expanding the employment and involvement of healthcare practitioners in the provision of mental healthcare services in the community improves the delivery of comprehensive care to MHCUs and having sufficient numbers of professionally trained healthcare practitioners could raise the standard of mental healthcare in the community. These measures will allow MHCUs to receive the attention they require:

‘Firstly, is to employ healthcare professionals who are well trained when it comes to psychiatry.’ (Participant C, female, RN)

The employment of more healthcare practitioners will alleviate the scarcity of healthcare practitioners and enhance the likelihood of a stable number of healthcare practitioners in the future (Shamsi & Peyravi 2020).

#### Subtheme 4.6: More in-service training for healthcare practitioners

Knowledge is the best tool, especially in the healthcare setting. For instance, in-service training for healthcare practitioners would facilitate better mental healthcare provision. As shared by participants, in-service training might help healthcare practitioners to improve their understanding of psychiatric or mental healthcare and the proper care of the MHCUs:

‘There should be more in-service training when it comes to psychiatry care I know we have done psych but we need training I believe there is new information coming every day.’ (Participant C, female, RN)

According to the literature, healthcare practitioners caring for MHCUs require substantial training, dedication and motivation to deliver comprehensive treatment (Alburquerque-Sendin et al. 2018; Joubert & Bhagwan 2018).

## Conclusion

The findings of this study provide light on the participants’ views on community-based comprehensive care and meet its aim and objectives. The participants argued that comprehensive care is necessary. According to healthcare practitioners, better coordination of mental healthcare services will improve psychiatric treatment and prevent relapses in MHCUs. To maintain comprehensive care, relevant stakeholders must be included. This study has resulted in recommendations for nursing practice, nursing education and nursing research.

### Limitation of the study

The intention was to include all members of the MDT in this study, but psychologists, social workers, psychiatrists and other psychiatric or mental healthcare practitioners were not available in community settings or clinics. To limit the risk of contributing to the spread of COVID-19, the latter practitioners were only based in hospitals. Therefore, only professional nurses and one medical doctor participated in data collection. These circumstances posed a challenge for data collecting and resulted in the researcher having to revise the data collection method from focus group interviews to telephonic semistructured individual interviews.

### Recommendations

The recommendations resulting from the study are aimed at improving comprehensive care through improving healthcare practitioners’ knowledge and insight into this topic. This can be done through workshops, training, advanced degrees and diplomas. Mental health campaigns, community outreach programmes and home visits should all be used to promote and maintain comprehensive care. For the efficient and smooth provision of comprehensive care, MDT members should be trained to function in community settings.

Research should be carried out on the perspectives of community members, caregivers and family members on adherence to mental health medication. Measures that obstruct service delivery related to mental health medication should be conducted and the findings applied. More research should be performed on the factors that contribute to retention and shortages of nurses.

Nursing practice should strive for the availability of mental health medication by ordering on time. Further, the Department of Health should hire more health professionals. There should be the creation and maintenance of community enrichment projects and starting up of support groups among the MHCUs.
